# Needle-free technique to prevent guidewire damage during endoscopic ultrasound-guided hepaticogastrostomy

**DOI:** 10.1055/a-2598-3933

**Published:** 2025-05-22

**Authors:** Takeshi Ogura, Jun Matsuno, Takafumi Kanadani, Ahmad Fikry Aboelezz, Hiroki Nishikawa

**Affiliations:** 1 Endoscopy Center, Osaka Medical and Pharmaceutical University Hospital, Takatsuki, Japan; 2 2nd Department of Internal Medicine, Osaka Medical and Pharmaceutical University, Takatsuki, Japan; 3 Department of Internal Medicine, Gastroenterology and Hepatology Unit, Tanta University, Tanta, Egypt

Endoscopic ultrasound-guided hepaticogastrostomy (EUS-HGS) is now widely performed for patients with duodenal obstruction or surgically altered anatomy. During EUS-HGS, guidewire insertion may be a challenging step, as previously reported 1 . To improve the technical success rate of guidewire manipulation without causing guidewire damage by shearing, the liver impaction technique can be helpful 2 3 . However, if the angle between the needle and the bile duct is acute, guidewire damage can occur, even if the liver impaction technique is used. To overcome this, we perform guidewire manipulation using a needle-free technique. Technical tips for the needle-free technique are presented.

An 80-year-old woman was admitted to our hospital for the treatment of obstructive jaundice caused by cancer of the head of the pancreas. After duodenal stent deployment, EUS-HGS was attempted. The intrahepatic bile duct was punctured using a 19-G needle, and contrast medium was injected. On cholangiography, it was seen that the angle between the biliary tract and the needle was acute. The 0.025-inch guidewire was therefore advanced into the periphery of the bile duct, but guidewire damage occurred again ( Fig. 1 **a** ). To manipulate the guidewire, the liver impaction technique was used, and the guidewire could be directed to the intrahepatic bile duct ( Fig. 1 **b** ). To advance the guidewire into the common bile duct, the guidewire was pulled back ( Fig. 1 **c** ), but guidewire shearing occurred again. Therefore, the sheath of the needle was pulled back into the EUS scope ( Fig. 1 **d** ). By doing so, the angle between the needle and the guidewire increased to 180 degrees, and the guidewire could be manipulated easily. After successful guidewire deployment within the common bile duct, a partially covered, self-expandable, metal stent was deployed without any adverse events ( Fig. 2 , Video 1 ).

**Fig. 1:**
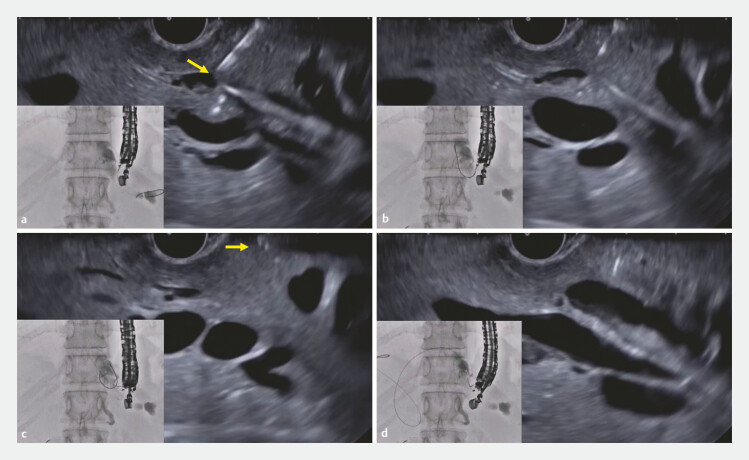
Endoscopic ultrasound images. **a** Guidewire damage occurred (arrow) during insertion. **b** After the liver impaction technique, guidewire advancement into the hepatic hilar site was performed successfully. **c** The needle was completely pulled within the sheath (arrow). **d** After the sheath was pulled back into the echoendoscope, guidewire deployment into the common bile duct was performed successfully.

**Fig. 2:**
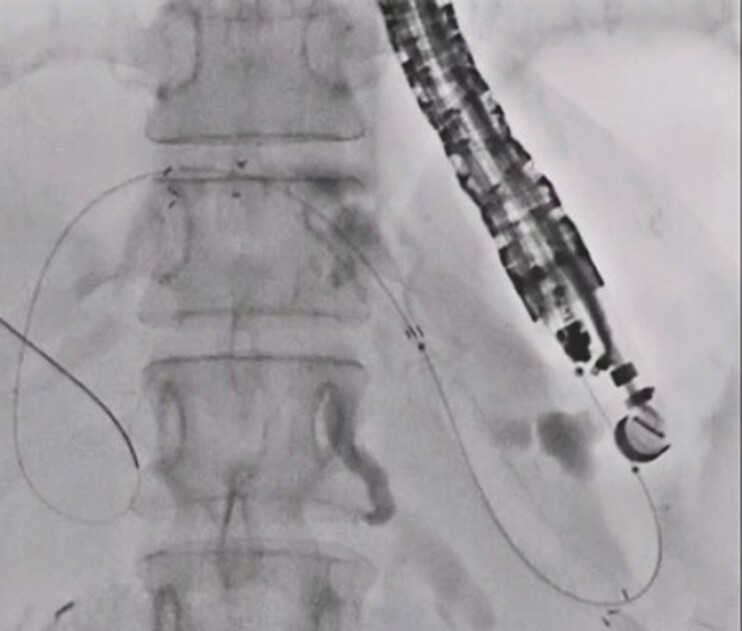
Metal stent deployment from the intrahepatic bile duct to the stomach was performed successfully.

Guidewire manipulation was performed using the needle-free technique.Video 1

In conclusion, in cases of difficult guidewire manipulation during EUS-HGS, the needle-free technique might be helpful to prevent guidewire damage and improve the technical success of guidewire manipulation.

Endoscopy_UCTN_Code_TTT_1AS_2AD

## Notes

Endoscopy E-Videos https://eref.thieme.de/e-videos
*E-Videos* is an open access online section of the journal *Endoscopy* , reporting on interesting cases and new techniques in gastroenterological endoscopy. All papers include a high-quality video and are published with a Creative Commons CC-BY license. Endoscopy E-Videos qualify for HINARI discounts and waivers and eligibility is automatically checked during the submission process. We grant 100% waivers to articles whose corresponding authors are based in Group A countries and 50% waivers to those who are based in Group B countries as classified by Research4Life (see: https://www.research4life.org/access/eligibility/ ). This section has its own submission website at https://mc.manuscriptcentral.com/e-videos .

## References

[1] Vila JJ Pérez-Miranda M Vazquez-Sequeiros E Initial experience with EUS-guided cholangiopancreatography for biliary and pancreatic duct drainage: a Spanish national survey Gastrointest Endosc 2012 76 1133 1141 23021167 10.1016/j.gie.2012.08.001

[2] Ogura T Masuda D Takeuchi T Liver impaction technique to prevent shearing of the guidewire during endoscopic ultrasound-guided hepaticogastrostomy Endoscopy 2015 47 E583 E584 26649471 10.1055/s-0034-1393381

[3] Nakamura J Ogura T Ueno S Liver impaction technique improves technical success rate of guidewire insertion during EUS-guided hepaticogastrostomy (with video) Therap Adv Gastroenterol 2023 16 17562848231188562 10.1177/17562848231188562 PMC10475223 37667804

